# Differential Modulation of 25-hydroxycholecalciferol on Innate Immunity of Broiler Breeder Hens

**DOI:** 10.3390/ani11061742

**Published:** 2021-06-10

**Authors:** Pao-Chia Chou, Pei-Chi Lin, Shu-Wei Wu, Chien-Kai Wang, Thau-Kiong Chung, Rosemary L. Walzem, Lih-Shiuh Lai, Shuen-Ei Chen

**Affiliations:** 1 Department of Food Science and Biotechnology, National Chung Hsing University, Taichung 40227, Taiwan; paochia88@yahoo.com.tw; 2 Department of Animal Science, National Chung Hsing University, Taichung 40227, Taiwan; bonnie800212@hotmail.com (P.-C.L.); wendypink9@hotmail.com (S.-W.W.); wchienkai@nchu.edu.tw (C.-K.W.); 3 The iEGG and Animal Biotechnology Center, National Chung Hsing University, Taichung 40227, Taiwan; 4 DSM Nutritional Products Asia Pacific, Singapore 117440, Singapore; Thau-Kiong.Chung@dsm.com; 5 Department of Poultry Science, Texas A&M University, College Station, TX 77843, USA; rwalzem@tamu.edu; 6 i-Center for Advanced Science and Technology (iCAST), National Chung Hsing University, Taichung 40227, Taiwan; 7 Innovation and Development Center of Sustainable Agriculture (IDCSA), National Chung Hsing University, Taichung 40227, Taiwan

**Keywords:** broiler breeder hens, 25-hydroxycholecalciferol, innate immunity, glucolipotoxicity, feed restriction

## Abstract

**Simple Summary:**

No predominant changes between R- vs. Ad-feed intake on leukocyte defense against pathogens were observed in broiler breeder hens despite some differences in inflammatory and respiratory burst responses. Overall, supplemental 25-OH-D_3_ had more pronounced effects on the innate immunity of Ad-hens. In vitro studies confirmed the differential effects of 25-OH-D_3_ to rescue immune functions altered by glucose and/or palmitic acid exposure.

**Abstract:**

Past immunological studies in broilers focused on juveniles within the rapid pre-slaughter growth period and may not reflect adult immune responses, particularly in breeders managed with chronic feed restriction (R). The study aimed to assess innate immune cell functions in respect to R vs. ad libitum (Ad) feed intake in breeder hens with and without dietary 25-hydroxycholecalciferol (25-OH-D_3_) supplementation. Ad-feed intake consistently suppressed IL-1β secretion, respiratory burst, and cell livability in peripheral heterophils and/or monocytes along the feeding trial from the age of 51 to 68 weeks. Supplemental 25-OH-D_3_ repressed IL-1β secretion and respiratory burst of both cells mostly in R-hens, but promoted monocyte phagocytosis, chemotaxis, and bacterial killing activity in Ad-hens in accompany with relieved hyperglycemia, hyperlipidemia, and systemic inflammation. Overnight cultures with leukocytes from R-hens confirmed the differential effects of 25-OH-D_3_ to rescue immune functions altered by glucose and/or palmitic acid exposure. Studies with specific inhibitors further manifested the operative mechanisms via glucolipotoxicity in a cell type- and function-dependent manner. The results concluded no predominant changes between R- vs. Ad-feed intake on leukocyte defense against pathogens despite some differential differences, but supplemental 25-OH-D_3_ exerts more pronounced effects in Ad-hens.

## 1. Introduction

Modern broilers can reach a market weight of ~2 kg within 35 days, half of the time needed by their ancestors 50 years ago. Genetic selection expedited growth, but also resulted in reproductive inefficacy, and increased susceptibility to sudden death syndrome, fatty liver syndrome, and obesity-related morbidities such as cardiomyopathy [1,2,3,4]. Feed restriction is typically used to achieve target bodyweight gain in broiler breeders and improve reproductive performance and livability by limiting obesity and related dysfunctions [1,2,3,4].

Several studies found that immunological response was also a trade-off for rapid growth selection [5,6,7,8]. Genetic selection for improved growth performance has resulted in a decline of adaptive immune responses co-incident with an increase in cellular immunity and inflammatory responses [8]. To date, most of the immunological studies in growth selected lines of chickens were performed in juvenile birds during the 6 weeks prior to slaughter when massive lean tissue gain occurs. However, the effect of chronic feed restriction on the ability to develop optimal immunity in response to vaccination or against pathogen infection in breeder chickens is sparsely studied.

Increased fat mass in obese patients can induce metabolic dysregulation. It has also been recognized that obesity development is tightly associated with inflammatory status, particularly in the adipose tissue [9,10]. The abnormal provocation of factors of innate and adaptive immune system by inflammation acts as a strong inducer in the development of type 2 diabetes mellitus (T2DM) [9,11]. Our previous studies showed many metabolic commonalties existing between obese (T2DM) and broiler breeder hens consuming feed to appetite. Indeed, we have shown that prolonged Ad-feed intake by broiler breeder hens causes rapid fat deposition and produces lipotoxicity and an inflammatory response arising from lipid dysregulation and associated changes in gene expression and signaling that results in impaired functions in ovarian follicles, circulating leukocytes, heart, and pancreatic β-cells [3,4,12,13,14,15].

Innate immunity functions as the frontline defense against pathogen invasions; moreover, it also directs cellular and humoral responses to eliminate pathogens. Neutrophils and macrophages are key cells in the innate immune response against invading pathogens. Both types of cells function to clear pathogens through their phagocytic capacity and generation of oxidants that kill engulfed microorganisms [12]. The clearance of pathogens and apoptotic cells by phagocytic cells plays an important role in resolving inflammatory responses, and impairments of these processes often result in a chronic inflammatory state [16,17]. 

In a variety of models, vitamin D and its receptor signaling play an anti-obesity and –inflammatory role in the development of T2DM and cardiac pathogenesis [18,19]. Vitamin D was shown to be a pivotal component of the monocyte/macrophage response to infection [20,21], inducing their antimicrobial activity. Vitamin D_3_ (VD_3_) supplementation not only reversed VD_3_ deficiency–induced inflammatory responses but also alleviated immunological inflammation caused by LPS (lipopolysaccharide) in table-egg type laying hens [22,23]. We found that dietary 25-hydroxycholecalciferol (25-OH-D_3_) supplementation improved cardiac health and rescued the livability in the breeder hens partially by ameliorating systemic and cardiac inflammation and fibrotic progression [24,25,26,27]. The purpose of the present studies was to assess the innate immune functions of breeder hens consuming feed to appetite (Ad) and hens fed to achieve target bodyweight by commercial feed restriction recommendations (R) as we found no literature on the topic. Besides, whether the beneficial effects of dietary 25-OH-D3 supplementation extended to innate immunological responses was evaluated in both R- and Ad-hens. In vitro studies with isolated heterophils and monocytes in combination with pharmacological inhibition in glucose and fatty acid metabolism were used to delineate the mechanisms of 25-OH-D_3_ on innate immune functions.

## 2. Materials and Methods

### 2.1. Animal Management

A flock of broiler breeder hens (Arbor Acres Plus FF) at age 23 weeks were purchased from a local breeder farm. Hens were caged individually and fed a standard soy and corn-based breeder layer mash (11.6 MJ/kg metabolizable energy; 16% crude protein) with weekly adjustment of feed allocation to achieve the targeted bodyweight as per recommendations [3,4]. The study was designed as a 2 × 2 Latin Square. At the age of 51 weeks, 48 birds were continued with restricted rations (R-hens, 150–160 g feed/day/hen) as recommended, while another group of 48 birds were allowed sufficient feed for consumption to appetite (Ad-hens) until 68 weeks of age. Within each feeding level (Ad or R), half of the hens (*n* = 24) were provided with the standard breeder diet supplemented with an additional 69 μg 25-OH-D_3_/kg feed (2760 IU/kg feed equivalent, DSM Nutritional Products Ltd., The Netherlands). All birds were maintained at ambient temperatures and humidities of 25–28 °C and 60–75%, respectively. Birds were provided with free access to water throughout the experiment. Feed was placed at 08:30 a.m. in conjunction with a 14L:10D (lights on at 05:00 a.m.) photoschedule. All bird husbandry and tissue collections were conducted in accordance with an approved animal care protocol of the National Chung Hsing University, Taiwan (IACUC Permit No. 102–113). Egg production and feed intake were recorded daily, and body weight was recorded each week.

### 2.2. Determination of Plasma Glucose, Triacylglycerol, NEFA, Insulin, and IL-1β Concentrations

Overnight fasting blood samples were collected from 6 randomly selected hens in each treatment group, consisting of hens at 58 and 65 weeks of age from a wing vein. On those same days, a second blood sample was collected 30 min after re-feeding 20 g of feed. Commercial kits were used to determine plasma triacylglycerol (TG, Sigma-Aldrich, St. Louis, MO, USA), glucose and NEFA (non-esterified fatty acid, Wako Chemicals, Osaka, Japan) levels. Plasma insulin and interleukin-1β (IL-1β) concentrations were determined by a validated commercial ELISA kit (Cat.# 10-1249-01, Uppsala, Sweden, KMC006; Biosource International, Camarillo, CA, USA, respectively) [28,29].

### 2.3. Isolation of Peripheral Leukocytes

Hens in the Ad-group that were 59–60 weeks old had consumed unrestricted amounts of feed for 8–9 weeks. In that same Ad-group, hens that were 66–68 weeks old had consumed unrestricted amounts of feed for 15–17 weeks. At each age, venous blood from two randomly selected hens was pooled for leukocyte isolation and functional analysis of freshly prepared cells. A total of six independent preparations were prepared for each diet group. For mechanistic studies, peripheral blood leukocytes were isolated from 61 to 65-week-old hens and used for overnight cultures with various treatments. In those mechanistic studies, blood from four hens was pooled prior to monocyte and heterophil isolation by discontinuous gradient centrifugation using commercial Histopaque^®^ 1077 and 1119, respectively (Sigma-Aldrich, St. Louis, MO, USA) [12,30,31]. A total of four independent preparations were analyzed for mechanistic studies.

### 2.4. Cell Cultures

Isolated heterophils (equivalent to neutrophils in mammals) or monocytes were cultured in RPMI-1640 medium (containing 10% fetal bovine serum, 50 units/mL penicillin G sodium and streptomycin, pH 7.4) at 37 °C, 5% CO_2_, 95% humidity for 3 h. Cells were then treated with 25-OH-D_3_ (dissolved in anhydrous ethanol, Sigma-Aldrich, St. Louis, MO, USA), glucose (in distilled water, Sigma-Aldrich, St. Louis, MO, USA), and/or palmitic acid (PA, Sigma-Aldrich, St. Louis, MO, USA) at the indicated concentrations within the physiological conditions for overnight [3,4,13,32]. Palmitic acid was supplemented as a complex with 5% fatty acid-free BSA (8:1, M:M) (Research Organics, Cleveland, OH, USA) [33,34]. Fatty acid free BSA-supplemented media was used as a control.

To ascertain the mechanisms of glucolipotoxicity, cells were pre-treated with an individual inhibitor of key steps in the metabolic processes identified for glucolipotoxicity. The inhibitory compounds (Sigma-Aldrich, St. Louis, MO, USA) were fumoninsin B1 (FB1), a ceramide synthase inhibitor in ceramide and sphingomyelin synthesis pathway; n-(2-mercaptopropionyl)-glycine (nMPG), a scavenger of reactive oxygen species (ROS); Desipramine (DPS), a functional inhibitor of acid sphingomyelinase that blocks sphingomyelin breakdown into ceramide; and Triacsin-C (TC), a pharmacologic inhibitor of long chain fatty acyl-CoA synthetase that blocks fatty acid activation and downstream metabolism. Inhibitor concentrations and exposure times varied for FB1: final concentration 25 µM for 1 h; nMPG, final concentration 0.3 mM for 30 min; DPS, final concentration 10 µM, for 2 h; and TC, final concentration 5 μM for 2 h. Fumoninsin B1, n-MPG, and DPS were dissolved in distilled water and TC was dissolved in DMSO (dimethyl sulfoxide). Compounds were added to the medium in a 1:1000 ratio (*vol*/*vol*). Following pre-treatment, medium was replaced with treatment media comprised of basal medium in the presence or absence of glucose or PA, or both at the indicated levels and the presence or absence of 25-OH-D_3_ for overnight (16 h) incubation. Media and cells were collected following incubation for further analyses.

### 2.5. Respiratory Burst and Phagocytosis Analysis

Collected cells were incubated with 1 mL of freshly made luminol (4 × 10^−4^ M in HBSS containing 0.05 M Na_2_CO_3_ and 1.5 mM CuSO_4_) in the presence of phorbol 12-myristate 13-acetate (PMA; final concentration 5 ng/mL; Sigma-Aldrich, St. Louis, MO, USA) stimulation. Generation of oxidative radicals was measured with luminol-dependent chemiluminescence by a Triathler luminometer (Hidex, Turku, Finland) [12,31,35]. Cellular phagocytic activity was measured by a zymosan-dependent method as described previously [12,31].

### 2.6. Cell Chemotaxis Analysis

Chemotaxis were analyzed by cell migration across the membrane according to the trans-well procedures developed previously with some modifications [31]. A total of 600 μL Roswell Park Memorial Institute (RPMI)-1640 medium containing 10% BSA was loaded in a 24-well plate. Approximately 150 μL RPMI-1640 medium treated cells (2 × 10^5^) were loaded into the cell culture inserts (5 μm, Millipore, Billerica, MA, USA). The inserts were put on the 24-well plate and incubated at 37 °C for 1 h. Unmigrated cells were removed by wiping the top surface of the membrane. The migrated cells present on the bottom surface of the membrane were fixed with methanol for 30 min, stained by Coomassie blue R250 (0.5% in 50% ethanol) for 30 min and destained in 10% acetic acid. After drying, the membrane was mounted onto a slide and migrated cells were counted (5 random fields per slide) in 400× fields. The number of migrated cells was enumerated by light microscopy.

### 2.7. Bacterial Killing Analysis

Bacterial killing activity was evaluated using a chicken nalidixic acid-resistant strain of virulent *Salmonella* *typhimurium* (ST, clone number #15721) [36]. In brief, ST was prepared by propagation in Luria Bertani (LB) broth (Acumedia, Lansing, MI, USA) at 37 °C for 12 h and an aliquot of broth was used for viable bacterial count using the LB agar plate. Freshly isolated peripheral heterophils or monocytes and opsonized ST were seeded in a well of microplate at 1:2 ratio (5 × 10^5^ cells: 1 × 10^6^ colony forming units, CFUs of ST) in duplicates. The plates were centrifuged (500× *g*, 5 min, 4 °C) and incubated at 41 °C with 5% CO_2_ for 45 min. After centrifugation and (1000× *g*, 5 min, 4 °C) and removal of the supernatants, cells were then lysed for 3 min after adding 100 μL of deionized water into the wells. After lysis, 10 μL of WST-8 (supplied in Cell Counting Kit-8, Sigma-Aldrich, St. Louis, MO, USA) were added to the wells for color development as instructed by the supplier. Optical density was read at 450 nm using a microtiter plate spectrophotometer. The method relies on the proportionality between viable bacteria conversion of WSTR-8 into water-soluble formazan dye. Wells seeded with opsonized ST only served as a positive control. The Cell Counting Kit-8 assay was validated by counting colony forming units from control and test wells, as described above. The killing activity was calculated as per the following formula;

%killing = (OD cell + OD bacterial − OD sample)/(OD cell + OD bacterial) × 100(1)

### 2.8. Cell Viability and Livability Analysis

Cells harvested after overnight cultures were used for viability analysis by Fluorescein (FITC)-Annexin-V/propidium iodide (PI) staining and cytometry sorting as described previously [13]. Results of early (defined as FITC-annexin V-positive and PI-negative), late (FITC-annexin V- and PI-positive) stage apoptosis, and necrosis (FITC-annexin V-negative and PI-positive staining) were pooled to calculate total cell death and viability. Freshly prepared leukocytes suspended in RPMI-1640 medium containing 10% BSA for 1 h were used for livability analysis based on the same FITC-Annexin-V/PI staining method.

### 2.9. Western Blot Analysis

Collected media were concentrated (approximately 20×) using a centrifugal concentrator with a molecular weight cut-off of 3 kDa (Millpore Merck, Darmstadt, German). Aliquots of collected cells and concentrated medium were used for total protein extraction using RIPA buffer and then for Western blot analysis, as described previously [12,14]. A rabbit anti-chicken IL-1β primary antibody (Abcam, Cambridge, UK) and antibodies cross-reactive to chicken antigens, including rabbit anti-VDR (vitamin D receptor, Cat.# GR37-100UGCN, Merck Billerica, MA, USA), anti-p65 (a subunit of NFκB, nuclear factor kappa B, Cat.# 8242), and β-actin (Cat.# 4967, Cell Signaling Technology, Beverly, MA, USA), and a horseradish peroxidase-conjugated secondary antibody (Cell Signaling Technology, Beverly, MA, USA) were used in Western blot study.

### 2.10. Statistics

Data from functional analyses with freshly prepared cells were analyzed by two-way ANOVA, in which feed intake (Ad or R) and 25-OH-D_3_ treatment were the classifying variables. Differences between group means were tested using Bonferroni corrected *t*-test when the main effect was significant. If an interaction between feed intake and 25-OH-D_3_ treatment was found, a mean comparison was performed. Data from mechanistic studies were analyzed using one-way ANOVA by Tukey multiple comparison test or *t*-test. Results were expressed as means ± SE. Mean differences were considered significant at *p* < 0.05. All statistical procedures were carried out using SPSS (Chicago, IL, USA) for Windows 13.0.

## 3. Results

### 3.1. Body Weight, Feed Intake, Plasma Glucose, Triglyceride, NEFA, Insulin, and IL-1β Levels

Regardless of 25-OH-D_3_ supplementation, Ad-feed intake showed an initial burst of feed consumption in the first week from the prescribed 157 to 196 g/day/hen. Feed intake then declined gradually to reach a nadir at 62 weeks (132 g/day/hen), and thereafter increased slowly to 153 g/day/hen at 68 weeks (Supplementary Figure S1A). The BW of Ad-hens increased sharply to reach 4.6 kg/hen at age of 60 weeks, declined slightly to 63–64 weeks, and subsequently, increased to reach 4.6–4.7 kg/hen at 68 weeks (Supplementary Figure S1B). This pattern of feed intake and BW gain was in marked contrast to a slow increase of BW from ~3.6 kg at 51 weeks to ~3.95 kg at 68 weeks with the breeder prescribed feed allotments.

**Figure 1:**
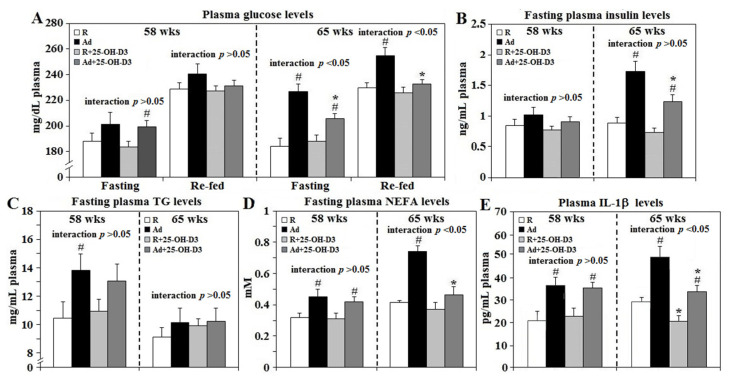
Plasma glucose, insulin, TG, NEFA, and IL-1β concentrations of broiler breeder hens provided with restricted (R) or ad libitum (Ad) feed intake. At age of 58 and 65 weeks (wks), 6 hens from each group were randomly selected for blood collection for plasma glucose, insulin, triacylglycerol (TG), non-esterified fatty acid (NEFA), and interleukin-1β (IL-1β) analysis (**A**–**E**, respectively, *n* = 6). #; significant difference by Ad-feed intake (vs. corresponding R hens, *p* < 0.05), *; significant difference by 25-OH-D_3_ (vs. R- or Ad-hens, *p* < 0.05). 25-OH-D_3_: 25-hydroxycholecalciferol.

Ad-feed intake provoked obesity-associated metabolic dysregulations including increased fasting plasma glucose levels and 30 min re-fed plasma glucose concentrations at 65 weeks. Increases in circulating insulin, TG, and NEFA concentrations were observed in Ad-hens at both 58 and 65 weeks (*p* < 0.05, Figure 1A–D). Dietary 25-OH-D_3_ supplementation suppressed plasma glucose, insulin and NEFA levels of Ad-hens at 65 weeks but did not affect concentrations in R-hens. Ad-feed intake also provoked chronic systemic inflammation as shown by increased plasma IL-1β concentrations at 58 and 65 weeks. Supplemental 25-OH-D_3_ significantly suppressed plasma IL-1β levels in both R- and Ad-hens at 65 weeks (*p* < 0.05, Figure 1E). A significant interaction between Ad and 25-OH-D_3_ treatments was found for plasma glucose, NEFA, and IL-1β level at 65 weeks (*p* < 0.05, Figure 1A–D) as 25-OH-D_3_ decreased values more in Ad-hens than in R-hens.

### 3.2. IL-1β Secretion, Phagocytosis, and Respiratory Burst of Fresh Leukocytes

**Figure 2:**
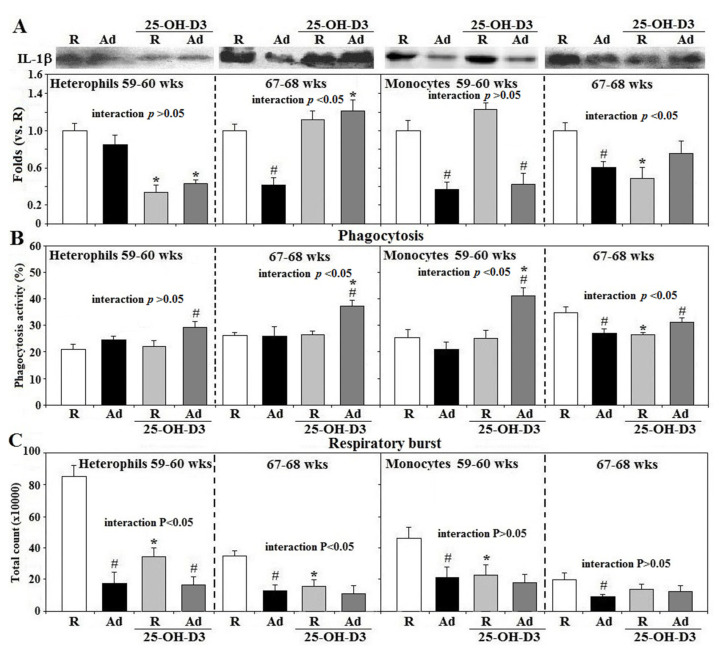
Effects of dietary supplementation of 25-OH-D_3_ on IL-1β secretion, phagocytosis, and the respiratory burst of leukocytes in broiler breeder hens provided with restricted (R) or ad libitum (Ad) feed intake. Peripheral leukocytes (2 × 10^6^ cells) isolated from hens at age 59–60 and 67–68 weeks (wks) were cultured for 3 h and medium were then collected for interleukin-1β (IL-1β) analysis (**A**) by Western blot method based on equivalent amounts of protein (*n* = 6). Freshly prepared cells were used for phagocytosis analysis (**B**) and respiratory burst analysis (**C**) (*n* = 6). Chemiluminescence results of respiratory burst analysis were expressed as integrated counts over 45 min. Results of Western blots were expressed as rations relative to R-hens. *; significant difference by 25-OH-D_3_, *p* < 0.05, #; significant difference by Ad-feed intake. 25-OH-D_3_; 25-hydroxycholecalciferol.

In contrast to R-hens, freshly isolated heterophils at 67–68 weeks and monocytes at both 59–60 and 67–68 weeks from Ad-hens had lower IL-1β secretion (*p* < 0.05, Figure 2A). Supplemental 25-OH-D_3_ suppressed heterophil IL-1β secretion in both R- and Ad-hens at 59–60 weeks and in Ad- and R-hen monocytes at 59–60 and 67–68 weeks, respectively, but increased heterophil IL-1β secretion in Ad-hens at 67–68 weeks. Ad-hen monocytes had a lower phagocytic activity at 67–68 weeks and supplemental 25-OH-D_3_ increased the phagocytosis of Ad-hen heterophils and monocytes at 67–68 weeks and at 59–60 weeks, respectively (*p* < 0.05, Figure 2B). Ad-feed intake also significantly suppressed respiratory burst response in both types of leukocytes at 59–60 and 67–68 weeks (*p* < 0.05), which 25-OH-D_3_ did not alter in Ad-hens. This same variable was significantly decreased in R-hen heterophils and monocytes at 59–60 and/or 67–68 weeks (*p* < 0.05, Figure 2C) with 25-OH-D_3_ supplementation. A significant interaction between Ad×25-OH-D_3_ treatments was observed in IL-1β secretion and phagocytosis of both cell types at 59–60 and/or 67–68 weeks, and in the respiratory burst response of heterophils at 59–60 and 67–68 weeks (*p* < 0.05, Figure 2A–C).

### 3.3. Chemotaxis and Bacterial Killing of Fresh Leukocytes

**Figure 3:**
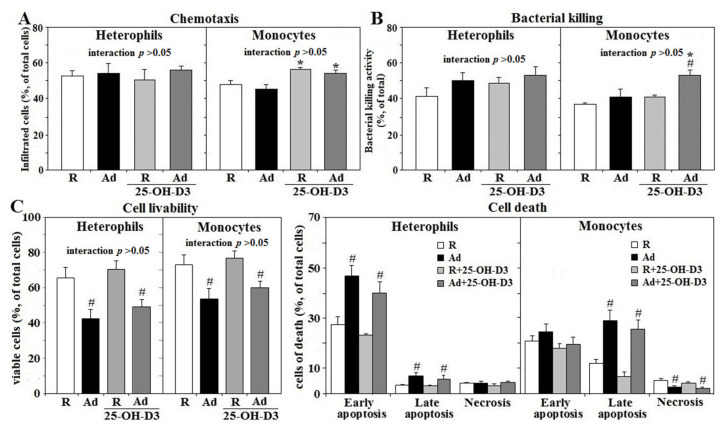
Effects of dietary supplementation of 25-OH-D_3_ on chemotaxis, bacterial killing and cell livability of leukocytes in broiler breeder hens under restricted (R) or ad libitum (Ad) feed intake. Freshly prepared cells (2 × 10^5^) from hens at age of 66 weeks were used for chemotaxis analysis through trans-well method (**A**), incubated with *Salmonella Typhimurium* (ST) at a 1:2 ratio for 45 min for bacterial killing analysis (**B**), or suspended in RPMI for cell livability analysis (**C**) (*n* = 6). Cell viability was defined as the percentage of total cells present that were viable in a cell death analysis with FITC-Annexin-V/propidium iodide (PI) staining and cytometry sorting. Results of bacterial killing were expressed as ratios relative to R-hens. *; significant difference by 25-OH-D_3_, *p* < 0.05, #; significant difference by Ad-feed intake. 25-OH-D_3_; 25-hydroxycholecalciferol.

Ad-feed intake exerted no significant effects on chemotaxis and bactericidal activity in either type of leukocytes. However, supplemental 25-OH-D_3_ increased monocyte chemotaxis in both Ad- and R-hens, as well as bactericidal activity in Ad-hens (*p* < 0.05, Figure 3A,B). Viability of both types of leukocytes was decreased in Ad-hens compared to those of R-hens. Early apoptosis accounted for most of the heterophil death in both R- and Ad-hens, whereas late apoptosis contributed most of the death in monocytes (*p* < 0.05, Figure 3C). Supplemental 25-OH-D_3_ had no effects on the viability of either cell type.

### 3.4. VDR Protein Amounts and NFκB Activation of Fresh Leukocytes

**Figure 4:**
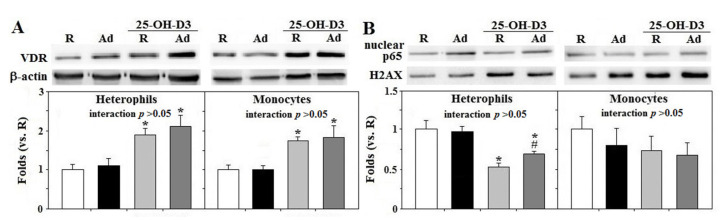
Effects of dietary supplementation of 25-OH-D_3_ on VDR protein amounts and p65 activation of leukocytes in broiler breeder hens provided with restricted (R) or ad libitum (Ad) feed intake. Freshly prepared cells from hens at the age of 66 weeks were used for total protein extraction for vitamin D receptor (VDR) expression (**A**) and nuclear extracts were used for p65 (a subunit of nuclear factor kappa B, NFκB) translocation (**B**) through Western blot method. Results were normalized to β-actin or H2AX and expressed as ratios relative to R-hens (*n* = 6). *; significant difference by 25-OH-D_3_, *p* < 0.05, #; significant difference by Ad-feed intake. 25-OH-D_3_; 25-hydroxycholecalciferol.

Ad-feed intake exerted no significant effects on VDR expression and p65 activation in both types of cells and supplemental 25-OH-D_3_ upregulated heterophil and monocyte VDR expression in both R- and Ad-hens, but suppressed heterophil p65 translocation (*p* < 0.05, Figure 4A–C). The results confirmed the inhibitory action of supplemental 25-OH-D_3_ on NFκB signaling in the animal model.

### 3.5. Effects of 25-OH-D_3_ on IL-1β Secretion of Leukocytes

**Figure 5:**
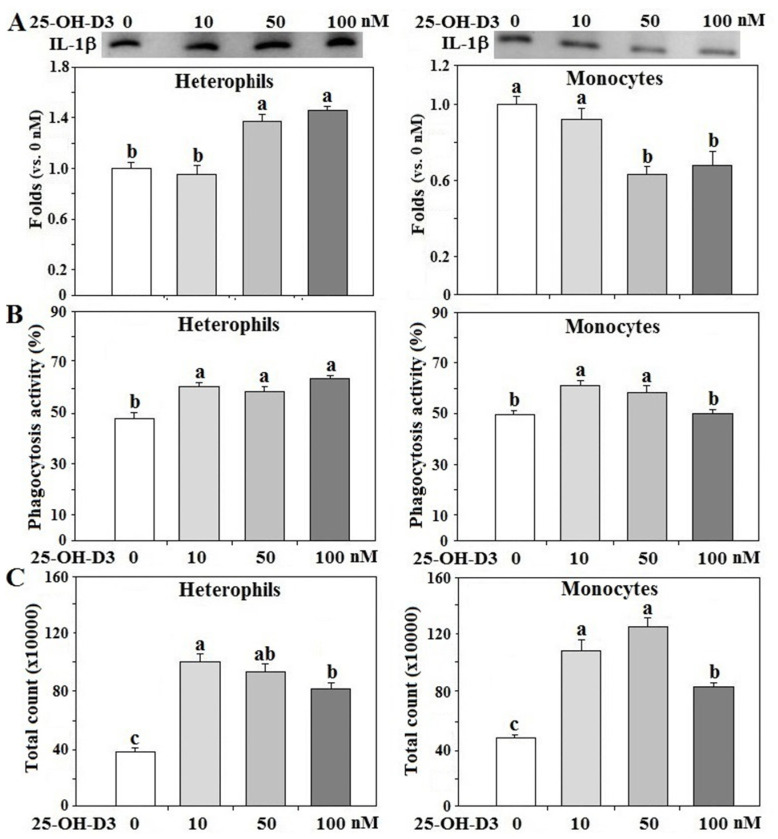
Effects of 25-OH-D_3_ on leukocyte functions. Peripheral heterophils and monocytes (2 × 10^6^ cells) isolated from R-hens at age 61–65 weeks were treated with various levels of 25-hydroxycholecalciferol (25-OH-D_3_) overnight (16 h). Media were collected for interleukin-1β (IL-1β) analysis by Western blot method based on protein equivalency (**A**); cells were collected for phagocytosis (**B**) and respiratory burst analysis (**C**) (*n* = 4). Chemiluminescence results of respiratory burst analysis were expressed as integrated counts over 45 min. Results of Western blots were expressed as ratios relative to control (0 nM of 25-OH-D_3_). Means with different letters differ significantly (*p* < 0.05).

In order to delineate the effects of 25-OH-D_3_ on innate immune functions of R- or Ad-fed hens, isolated leukocytes from R-hens were used for overnight culture studies. Treatment with 25-OH-D_3_ increased IL-1β secretion in heterophils in a dose-dependent manner, but decreased IL-1β secretion in monocytes (*p* < 0.05, Figure 5A). 25-OH-D_3_ differentially promoted phagocytosis and respiratory burst response in both types of leukocytes (*p* < 0.05, Figure 5B,C). Based on these results, the dose for 25-OH-D_3_ was optimized at 50 nM for the following studies.

### 3.6. Effects of Glucose and Fatty Acid on Leukocyte Functions

**Figure 6:**
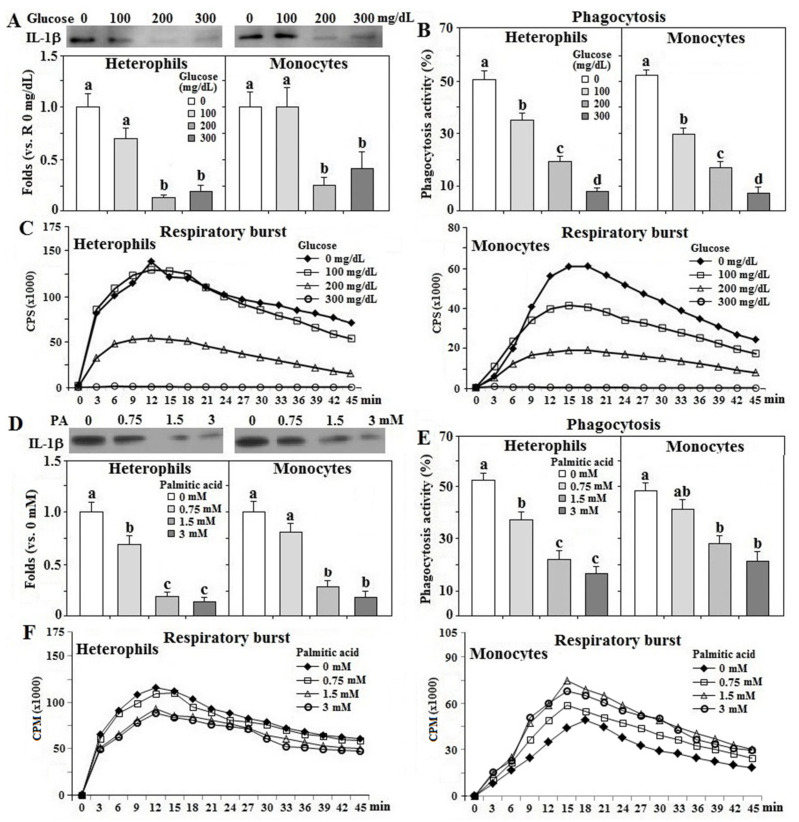
Effects of glucose and palmitic acid on leukocyte functions. Peripheral heterophils and monocytes (2 × 10^6^ cells) isolated from R-hens at age 61–65 weeks were treated with various levels of glucose or palmitic acid (PA) overnight (16 h). Media were collected for interleukin-1β (IL-1β) analysis by Western blot method based on equivalent amounts of protein (**A**,**D**) and collected cells were used for phagocytosis analysis (**B**,**E**) and respiratory burst analysis (**C**,**F**) (*n* = 4). Chemiluminescence results of respiratory burst analysis were expressed as count per second (CPM) over 45 min. Results of Western blots were expressed as rations relative to control (no glucose or PA supplementation). Means with different letters differ significantly (*p* < 0.05).

Treatment with glucose suppressed IL-1β secretion, phagocytosis, and respiratory burst response in both types of leukocytes in a dose-dependent manner (*p* < 0.05, Figure 6A–C). Since the glucose treatment at 300 mg/dL completely blocked the respiratory burst response in heterophils, and glucose at 100 mg/dL failed to affect IL-1β secretion, the dose for glucose effects in the mechanistic studies was optimized at 200 mg/dL. A dose-dependent suppression of IL-1β secretion and phagocytic activity by PA treatment was also observed in both cell types (*p* < 0.05, Figure 6D,E). Interestingly, PA treatment suppressed heterophil respiratory burst in a dose-dependent fashion, but increased the response in monocytes (*p* < 0.05, Figure 6F). The dose for PA effects on following studies was optimized at 1.5 mM.

### 3.7. Effects of 25-OH-D_3_ on Leukocyte Functions during Glucolipotoxicity

**Figure 7:**
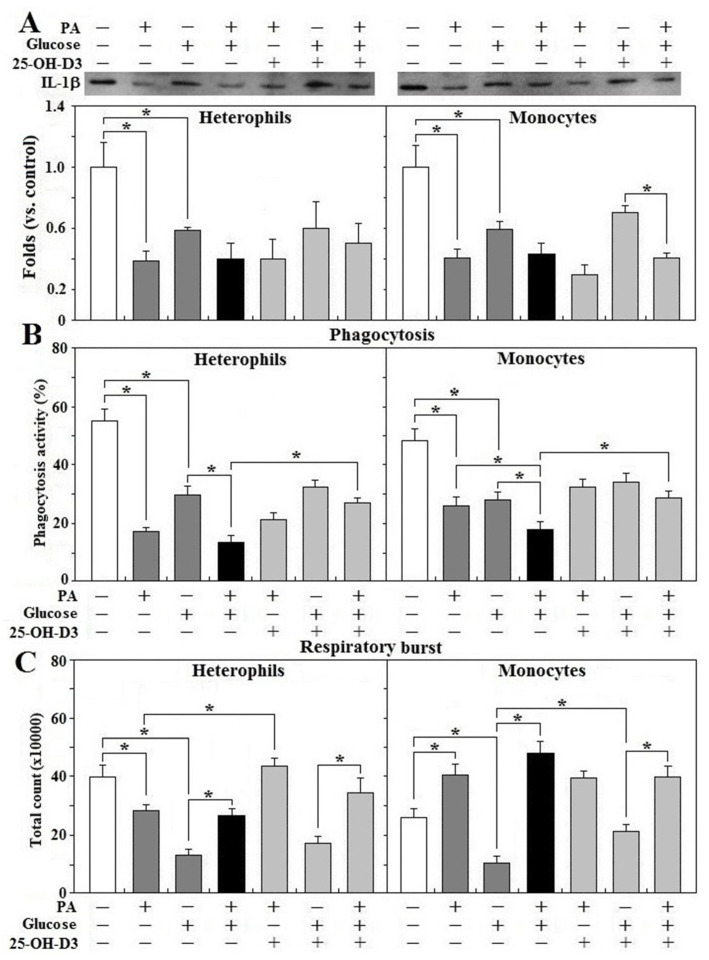
Effects of 25-OH-D_3_ on leukocyte functions challenged with glucose and/or palmitic acid. Peripheral heterophils and monocytes (2 × 10^6^ cells) isolated from R-hens at age 61–65 weeks were treated with vehicle, 25-hydroxycholecalciferol (25-OH-D_3_, 50 nM), palmitic acid (PA, 1.5 mM), and/or glucose (200 mg/dL) overnight (16 h). Media were collected for interleukin-1β (IL-1β) analysis (**A**) based on the equivalent amounts of protein and cells used for phagocytosis (**B**) and respiratory burst analysis (**C**) (*n* = 4). Chemiluminescence results of respiratory burst analysis were expressed as integrated counts over 45 min. Results of Western blots were expressed as rations relative to control (no PA, glucose and 25-OH-D_3_ supplementation). *, significant difference vs. corresponding control (*p* < 0.05). + or − indicates with or without PA, glucose, or 25-OH-D_3_.

Treatment of glucose or PA alone impaired IL-1β secretion in both heterophils and monocytes (*p* < 0.05) and combination of glucose and PA did not exacerbate the impairment (Figure 7A). It was shown that 25-OH-D_3_ had no effects on IL-1β secretion impaired by glucose, PA alone, or by their combination in either cell type. In the presence of 25-OH-D_3_, but not its absence, PA treatment further reduced IL-1β secretion caused by monocyte treatment with glucose (*p* < 0.05, Figure 7A). Combination of glucose and PA exacerbated phagocytic activity impaired by glucose or PA alone in both types of leukocytes and treatment of 25-OH-D_3_ rescued phagocytosis impaired by glucose and PA combination (*p* < 0.05, Figure 7B). In heterophils, glucose or PA alone impaired respiratory burst, while the addition of PA to the glucose treatment rescued the response impaired by glucose (*p* < 0.05, Figure 7C). In fact, 25-OH-D_3_ completely reversed heterophil respiratory burst suppressed by PA but not that caused by glucose alone or the glucose + PA treatment. Further, PA treatment promoted heterophil respiratory burst in the presence of glucose and 25-OH-D_3_ (*p* < 0.05, Figure 7C). In monocytes, glucose treatment impaired, but PA promoted respiratory burst response. Treatment with the combination of glucose and PA completely reversed the response impaired by glucose (*p* < 0.05, Figure 7C). Treatment with 25-OH-D_3_ significantly rescued the monocyte respiratory burst suppressed by glucose alone, but had no effects in the presence of PA. Interestingly, PA treatment significantly increased respiratory burst in both the glucose alone and glucose + 25-OH-D_3_ treatments (*p* < 0.05, Figure 7C).

### 3.8. Mechanisms of Gluco/Lipotoxicity on Leukocyte Functions

**Figure 8:**
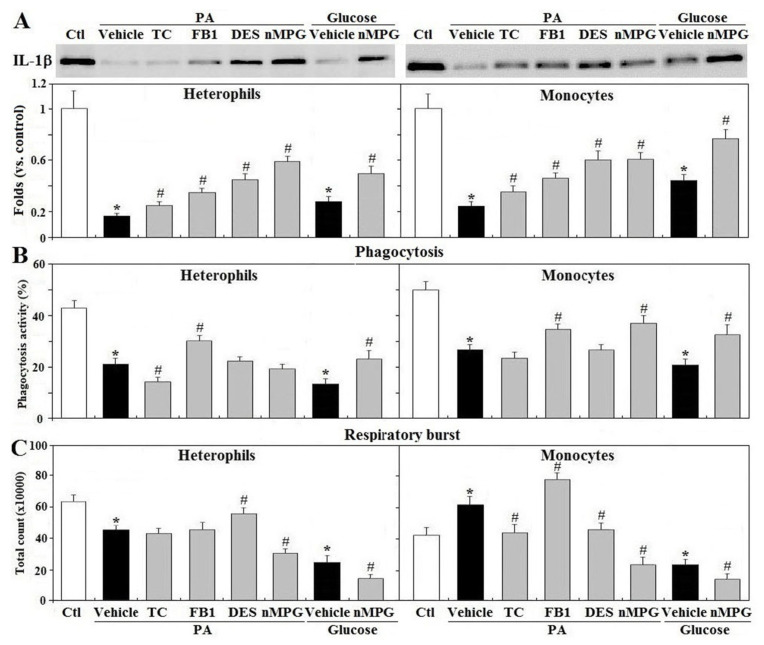
Mechanisms of glucolipotoxicity of leukocyte functions. Peripheral leukocytes (2 × 10^6^ cells) from R-hens at age 61–65 weeks were pre-treated with TC (Triacsin C, 5 μM), FB1 (fumonisin B1, 25 μM), DPS (Desipramine, 10 μM), or nMPG (N-mercaptopropionyl-glycine, 0.3 mM). After being replaced with the medium, cells were treated with the vehicle, palmitic acid (PA, 1.5 mM), or glucose (200 mg/dL) overnight (16 h). Media were collected for interleukin-1β (IL-1β) analysis (**A**) by Western blot method based on equivalent amounts of protein and collected cells used for phagocytosis (**B**) and respiratory burst analysis (**C**) (*n* = 4). Chemiluminescence results of respiratory burst analysis were expressed as integrated counts over 45 min. Results of Western blots were expressed as rations relative to control (Ctl). *; significant difference vs. corresponding control (vehicle), *p* < 0.05, #; significant difference vs. vehicle, *p* < 0.05.

In both types of cells, treatment with TC, a pharmacologic inhibitor of long chain fatty acyl-CoA synthetase that blocks fatty acid activation and downstream metabolism, FB1, the ceramide synthase inhibitor within the ceramide and sphingomyelin synthesis pathway, DPS, a functional inhibitor of acid sphingomyelinase that blocks sphingomyelin breakdown into ceramide, and nMPG that acts as a ROS scavenger, differentially rescued IL-1β secretion suppressed by PA or glucose (*p* < 0.05, Figure 8A). TC treatment exacerbated PA mediated suppression of phagocytosis in heterophils but not in monocytes, whereas FB1 partially rescued phagocytosis in both types of cells (*p* < 0.05, Figure 8B). Treatment with nMPG relieved phagocytosis impairment by PA or glucose in both types of leukocytes (*p* < 0.05, Figure 8B). In heterophils, DPS partially relieved respiratory burst suppressed by PA, whereas nMPG exacerbated PA or glucose mediated impairments (*p* < 0.05, Figure 8C). Treatment with TC or DPS reversed the PA-mediated increase in monocyte respiratory burst, while FB1 potentiated the increase by PA (*p* < 0.05, Figure 8C). nMPG significantly suppressed the promotion of respiratory burst by PA in monocytes to a level even lower than the control, and further exacerbated the suppression of respiratory burst by glucose (*p* < 0.05, Figure 8C).

### 3.9. Effects of 25-OH-D_3_ on Leukocyte Viability Following Glucose or Palmitic Acid Challenge

**Figure 9:**
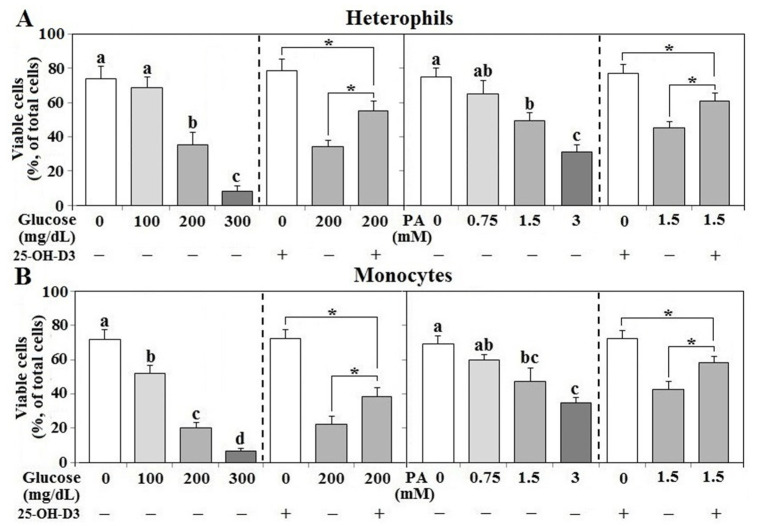
Effects of 25-OH-D_3_ on glucose or palmitic acid challenged leukocyte survival. Peripheral leukocytes (1 × 10^6^ cells) isolated from R-hens at age 61–65 weeks were cultured with indicated levels of glucose or palmitic acid (PA) in the presence or absence of 25-hydroxycholecalciferol (25-OH-D_3_, 50 nM) overnight (16 h). Collected cell were used for cell death analysis (*n* = 4). Means with different letters differ significantly among different levels of glucose or PA (*p* < 0.05). Means with different letters differ significantly (*p* < 0.05). *, significant difference vs. corresponding control (*p* < 0.05). Means with different letters differ significantly (*p* < 0.05). + or − indicates with or without PA, glucose, or 25-OH-D_3_. Figure 9 is for cell death analysis, (**A**,**B**) mean different cell types, (**A**) heterophils, (**B**) mpnocytes.

In both types of leukocytes, treatment of glucose or PA suppressed cell viability in a dose-dependent manner and 25-OH-D_3_ partially rescued cell survival (*p* < 0.05, Figure 9A,B). However, 25-OH-D_3_ alone had no significant effects on cell viability in either cell type.

## 4. Discussion

Very few studies have examined immune responses of broiler breeder hens, particularly with a view to compare hens reared with a restricted feeding regimen compared to those provided with unlimited access to feed. Consistent with our previous reports [14,24,25,26], Ad-feed intake provoked obesity-associated metabolic derangements including hyperglycemia, hyperlipidemia, and systemic inflammation. In humans and mice, the development of obesity and T2DM was suggested to associate with several defects in the innate immune responses, including decreased chemotaxis, phagocytosis and antimicrobial mechanisms in leukocytes [37,38]. Mice with T2DM were more susceptible to subcutaneous infection with *B. pseudomallei* and the increased severity of infection was associated with a higher expression of proinflammatory cytokines in paralleled with a rapid drop of blood glucose levels, as is frequently observed in sepsis. A failure of early immune responses to limit dissemination of infection and septic progression with fatal outcome in diabetic mice was primarily attributed to decreased phagocytic and antimicrobial activities in macrophages independent of neutrophil and dendritic cell functionality [38]. Ad-hens showed significantly diminished innate immune responses including impaired IL-1β secretion, respiratory burst, and cell viability in peripheral heterophils and monocytes. These results, particularly in heterophils, were confirmed by our previous studies using the same strain of breeder hens at younger ages (26–35 weeks) under Ad-feed intake for 3–8 weeks [12]. Interestingly, however, the present in vitro studies of immune cells isolated from Ad-hens showed no pronounced differences in chemotaxis, bacterial killing, and phagocytic activity compared to those isolated from R-hens. Accordingly, we concluded that only modest changes occurred in Ad-hen leukocyte defense against pathogens despite some differential differences in inflammation and respiratory burst responses. This is the first report to address the innate immune defense of broiler breeder hens managed with chronic feed restriction in comparison to hens allowed to consume feed to appetite. In a longitudinal study from age of 20 to 60 weeks, infectious causes by bacteria accounted for over 50% of the mortality in a broiler breeder flock [39]. While incompletely described, the commercially reared flocks studied would be expected to be managed with restrictive feeding. In vivo studies, therefore, are required to validate the response of innate immune system against infection of pathogens.

Excess of glucose and saturated fatty acids provoke a variety of cellular dysfunctions including cell death, ER (endoplasmic reticulum) stress, autophagy, insulin signaling, and inflammation, namely, glucolipotoxicity [40,41,42]. Type 2 diabetes is characterized by hyperglycemia, dyslipidemia, and increased inflammatory tone [43]. The stimulatory effects of hyperglycemia are enhanced by saturated fatty acids by engaging Toll-Like Receptor 2 (TLR2) and TLR4 receptors to induce ROS production, NFκB activation, and proinflammatory factor release in the adipose tissue and other tissues including leukocytes of the innate immune system to contribute to systemic inflammation and exacerbation of metabolic derangements as obesity and T2DM progress [43,44]. Consistent with our earlier reports, Ad-feed intake increased circulating glucose, insulin, NEFA, TG, and IL-1β concentrations. Surprisingly, however, peripheral heterophils and monocytes from Ad-hens had consistently lower IL-1β secretion in culture. Reduced secretion of IL-1β in the obese chicken model used here (i.e., Ad-hens) contrasts sharply with most reports in models of mammalian obesity, T2DM, and cellular models of glucolipotoxocity [40,42,43,44,45]. We further validated the observations with cell culture studies that used leukocytes from R-hens treated with glucose and/or palmitic acid within physiological levels [20] and again observed a suppressed inflammatory response in these leukocytes, including other functions such as respiratory burst in Ad-hens. Therefore, the innate immune cells apparently contribute little to the well documented systemic elevation in proinflammatory cytokines found in the adipose tissue, liver, heart, and ovary of the obese chicken model used here [14,24,26]. 

The combination of LPS and palmitate led to a synergistic increase of cellular ceramide in macrophages that augmented proinflammatory cytokine production of metabolic diseases characterized by dyslipidemia [46]. In addition to inflammatory response, ceramide and sphingomyelin metabolism play a critical role in modulating leukocyte functions including chemotaxis, respiratory burst, phagocytosis, and apoptosis [47,48,49,50,51]. The present results confirmed the development of glucolipotoxicity in the leukocytes of overfed chickens, in which palmitate and glucose differentially altered leukocyte functions that could subsequently be rescued by specific pharmacological inhibitors related to ceramide and/or ROS production. Since TC treatment only partially rescued the functions altered by PA, PA per se without activation of downstream metabolism via coenzyme A activation also contributed to lipotoxic development, possibly by interacting with a TLR to activate downstream signaling [42,43,44]. Among the assessed leukocyte functions, TC, FB1, DPS, or nMPG treatment rescued IL-β secretion suppressed by PA. Differential effects were observed for phagocytosis and respiratory burst activities depending on the function and leukocyte type. For example, PA treatment promoted respiratory burst in monocytes, whereas it suppressed this function in heterophils. The differential effects by PA alone or in combination with the specific inhibitors suggest an intrinsic difference in PA metabolism in TG storage, β-oxidation, and ceramide/sphingomyelin synthesis, by which heterophils apparently are more susceptible to lipotoxicity than monocytes. The suggestion was confirmed by differential ceramide and sphingomyelin contents of fresh heterophils and monocytes from R- and Ad-hens [12].

In innate immunity, vitamin D modulates both leukocyte and barrier cell functions to combat with pathogen infection [52,53]. It enhances antimicrobial peptide production, TLR expression and signaling, chemotaxis, and phagocytic activity. The anti-mycobacterial activity of 1α,25(OH)_2_D_3_ (1α,25 dihydroxycholecalciferol) is mediated by NADPH-dependent oxidase of phagocytes through phosphatidylinositol 3-kinase signaling [52,53]. Vitamin D also regulates the inflammatory states in the local microenvironments of infected sites by upregulating MAP kinase signaling and IL-4 expression and suppressing NF-kB activation and production of chemokines, proinflammatory cytokines and prostaglandin in both infiltrated leukocytes and damaged cells to resolve the inflammatory responses [54,55,56,57,58]. Previously, we reported that dietary 25-OH-D_3_ supplementation improved cardiac health and rescued the livability in breeder hens by ameliorating systemic hypoxia, hypertension, vascular remodeling, systemic and cardiac inflammation and fibrotic progression, and thereby alleviating pathological remodeling and functional compromise [24,25,26,27]. The present results further showed that supplemental 25-OH-D_3_ repressed IL-1β secretion and respiratory burst of both heterophils and monocytes primarily in R-hens, but promoted monocyte phagocytosis, chemotaxis, and bacterial killing activity in Ad-hens. The differential effects of 25-OH-D_3_ to rescue innate immune functions, particularly phagocytic activity, were further confirmed in the in vitro model using R-hen leukocytes in response to glucose and/or palmitic acid within physiological levels.

## 5. Conclusions

No predominant changes between R-hens vs. Ad-hens on leukocyte functions against pathogens in vitro were observed in broiler breeder hens despite some differences in inflammatory and respiratory burst response. Overall, supplemental 25-OH-D_3_ had more pronounced effects on the innate immunity of Ad-hens. In vitro studies showed detrimental effects of glucose and/or palmitic acid exposure on leukocyte functionality in a cell type- and function-dependent manner and further confirmed the differential effects of 25-OH-D_3_ to rescue the alterations of innate immune functions due to glucolipotoxicity.

## Supplementary Materials

The following are available online at https://www.mdpi.com/article/10.3390/ani11061742/s1, Figure S1: Effects of dietary supplementation of 25-OH-D_3_ on feed intake and body weight of broiler breeder hens provided with restricted or ad libitum feed intake.

## References

[1] Griffin H.D., Goddard C. (1994). Rapidly growing broiler (meat-type) chickens: Their origin and use for comparative studies of the regulation of growth. Int. J. Biochem..

[2] Yu M.W., Robinson F.E., Etches R.J. (1992). Effect of feed allowance during rearing and breeding on female broiler breeders. 3. Ovarian steroidogenesis. Poult. Sci..

[3] Chen C.Y., Lin H.Y., Chen Y.W., Ko Y.J., Liu Y.J., Chen Y.H., Walzem R.L., Chen S.E. (2017). Obesity-associated cardiac pathogenesis in broiler breeder hens: Pathological adaption of cardiac hypertrophy. Poult. Sci..

[4] Chen C.Y., Huang Y.F., Ko Y.J., Liu Y.J., Chen Y.H., Walzem R.L., Chen S.E. (2017). Obesity-associated cardiac pathogenesis in broiler breeder hens: Development of metabolic cardiomyopathy. Poult. Sci..

[5] Zou A., Nadeau K., Wang P.W., Lee J.Y., Guttman D.S., Sharif S., Korver D.R., Brumell J.H., Parkinson J. (2020). Accumulation of genetic variants associated with immunity in the selective breeding of broilers. BMC Genet..

[6] Willson N.L., Forder R.E.A., Tearle R.G., Nattrass G.S., Hughes R.J., Hynd P.I. (2017). Evaluation of fatty acid metabolism and innate immunity interactions between commercial broiler, F1 layer × broiler cross and commercial layer strains selected for different growth potentials. J. Anim. Sci. Biotechnol..

[7] Swaggerty C.L., Pevzner I.Y., Kaiser P., Kogut M.H. (2008). Profiling pro-inflammatory cytokine and chemokine mRNA expression levels as a novel method for selection of increased innate immune responsiveness. Vet. Immunol. Immunopathol..

[8] Cheema M.A., Qureshi M.A., Havenstein G.B. (2003). A comparison of the immune response of a 2001 commercial broiler with a 1957 randombred broiler strain when fed representative 1957 and 2001 broiler diets. Poult. Sci..

[9] Zhou T., Hu Z., Yang S., Sun L., Yu Z., Wang G. (2018). Role of adaptive and innate immunity in Type 2 Diabetes Mellitus. J. Diabetes Res..

[10] Lee C.H., Lam K.S. (2019). Obesity-induced insulin resistance and macrophage infiltration of the adipose tissue: A vicious cycle. J. Diabetes Investig..

[11] Richardson V.R., Smith K.A., Carter A.M. (2013). Adipose tissue inflammation: Feeding the development of type 2 diabetes mellitus. Immunobiology.

[12] Liu Z.C., Xie Y.L., Chang C.J., Su C.M., Chen Y.H., Huang S.Y., Walzem R.L., Chen S.E. (2014). Feed intake alters immune cell functions and ovarian infiltration in broiler hens: Implications for reproductive performance. Biol. Reprod..

[13] Xie Y.L., Pan Y.E., Chang C.J., Tang P.C., Huang Y.F., Walzem R.L., Chen S.E. (2012). Palmitic acid in chicken granulosa cell death-lipotoxic mechanisms mediate reproductive inefficacy of broiler breeder hens. Theriogenology.

[14] Pan Y.E., Liu Z.C., Chang C.J., Xie Y.L., Chen C.Y., Chen C.F., Walzem R.L., Chen S.E. (2012). Ceramide accumulation and up-regulation of proinflammatory interleukin-1β exemplify lipotoxicity to mediate declines of reproductive efficacy of broiler hens. Domest. Anim. Endocrinol..

[15] Huang Y.F., Chang L.C., Chen C.Y., Chen Y.H., Walzem R.L., Chen S.E. (2020). Unrestricted Feed Intake Induces β-Cell Death and Impairs Insulin Secretion in Broiler Breeder Hens. Animals.

[16] Chen L., Eapen M.S., Zosky G.R. (2017). Vitamin D both facilitates and attenuates the cellular response to lipopolysaccharide. Sci. Rep..

[17] Ambrose C.T. (2006). The Osler slide, a demonstration of phagocytosis from 1876 Reports of phagocytosis before Metchnikoff’s 1880 paper. Cell. Immunol..

[18] Vanlint S. (2013). Vitamin D and obesity. Nutrients.

[19] Pilz S., Tomaschitz A., Drechsler C., Dekker J.M., Marz W. (2010). Vitamin D deficiency and myocardial diseases. Mol. Nutr. Food Res..

[20] Hewison M. (2010). Vitamin D and the intracrinology of innate immunity. Mol. Cell. Endocrinol..

[21] Teymoori-Rad M., Shokri F., Salimi V., Marashi S.M. (2019). The interplay between vitamin D and viral infections. Rev. Med. Virol..

[22] Klebanoff S.J. (2005). Myeloperoxidase: Friend and foe. J. Leukoc. Biol..

[23] Geng Y., Ma Q., Wang Z., Guo Y. (2018). Dietary vitamin D3 supplementation protects laying hens against lipopolysaccharide-induced immunological stress. Nutr. Metab..

[24] Chou P.C., Chen Y.H., Chung T.K., Walzem R.L., Lai L.S., Chen S.E. (2020). Supplemental 25-hydroxycholecalciferol Alleviates Inflammation and Cardiac Fibrosis in Hens. Int. J. Mol. Sci..

[25] Lin H.Y., Chung T.K., Chen Y.H., Walzem R.L., Chen S.E. (2019). Dietary supplementation of 25-hydroxycholecalciferol improves livability in broiler breeder hens. Poult. Sci..

[26] Lin H.Y., Chou P.C., Chen Y.H., Lai L.S., Chung T.K., Walzem R.L., Huang S.Y., Chen S.E. (2019). Dietary supplementation of 25-hydroxycholecalciferol Improves livability in broiler breeder hens-amelioration of cardiac pathogenesis and hepatopathology. Animals.

[27] Yeh Y.L., Chou P.C., Chen Y.H., Lai L.S., Chung T.K., Walzem R.L., Huang S.Y., Chen S.E. (2020). Dietary supplementation of 25-hydroxycholecalciferol improves cardiac function and livability in broiler breeder hens–amelioration of blood pressure and vascular remodeling. Poult. Sci..

[28] Franssens L., Lesuisse J., Wang Y., De Ketelaere B., Willems E., Koppenol A., Guo X., Buyse J., Decuypere E., Everaert N. (2015). Prenatal tolbutamide treatment alters plasma glucose and insulin concentrations and negatively affects the postnatal performance of chickens. Domest. Anim. Endocrinol..

[29] Sevimli A., Misirlioglu D., Yagci A., Bulbul A., Yilmaztepe A., Altunbas K. (2008). The role of chicken IL-1β, IL-6 and TNF-α in the occurrence of amyloid arthropathy. Vet. Res. Commun..

[30] Kogut M.H., Genovese K.J., Lowry V.K. (2001). Differential activation of signal transduction pathways mediating phagocytosis, oxidative burst, and degranulation by chicken heterophils in response to stimulation with opsonized Salmonella enteritidis. Inflammation.

[31] Liu Z.C., Su C.M., Xie Y.L., Chang C.J., Chen J.Y., Wu S.W., Chen Y.H., Walzem R.L., Huang S.Y., Chen S.E. (2016). Intracellular lipid dysregulation interferes with leukocyte function in the ovaries of meat-type hens under unrestricted feed intake. Anim. Reprod. Sci..

[32] Chen S.E., McMurtry J.P., Walzem R.L. (2006). Overfeeding-Induced ovarian dysfunction in broiler breeder hens Is associated with lipotoxicity. Poult. Sci..

[33] Osawa Y., Uchinami H., Bielawski J., Schwabe R.F., Hannun Y.A., Brenner D.A. (2005). Roles for C16-ceramide and sphingosine 1-phosphate in regulating hepatocyte apoptosis in response to tumor necrosis factor-alpha. J. Biol. Chem..

[34] Spector A.A. (1986). Structure and lipid binding properties of serum albumin. Meth. Enzymol..

[35] Hala K., Moore C., Plachy J., Kaspers B., Bock G., Hofmann A. (1998). Genes of chicken MHC regulate the adherence activity of blood monocytes in Rous sarcomas progressing and regressing lines. Vet. Immunol. Immunopathol..

[36] Chuammitri P., Redmond S.B., Kimura K., Andreasen C.B., Lamont S.J., Palic D. (2011). Heterophil functional responses to dietary immunomodulators vary in genetically distinct chicken lines. Vet. Immunol. Immunopathol..

[37] Geerlings S.E., Hoepelman A.I. (1999). Immune dysfunction in patients with diabetes mellitus (DM). FEMS Immunol. Med. Microbiol..

[38] Hodgson K.A., Morris J.L., Feterl M.L., Govan B.L., Ketheesan N. (2011). Altered macrophage function is associated with severe Burkholderia pseudomallei infection in a murine model of type 2 diabetes. Microbes Infect..

[39] Naundrup Thøfner I.C., Poulsen L.L., Bisgaard M., Christensen H., Olsen R.H., Christensen J.P. (2019). Longitudinal Study on Causes of Mortality in Danish Broiler Breeders. Avian Dis..

[40] Lenin R., Maria M.S., Agrawal M., Balasubramanyam J., Mohan V., Balasubramanyam M. (2012). Amelioration of glucolipotoxicity-induced endoplasmic reticulum stress by a “chemical chaperone” in human THP-1 monocytes. Exp. Diabetes Res..

[41] Restaino R.M., Deo S.H., Parrish A.R., Fadel P.J., Padilla J. (2017). Increased monocyte-derived reactive oxygen species in type 2 diabetes: Role of endoplasmic reticulum stress. Exp. Physiol..

[42] Prieur X., Roszer T., Ricote M. (2010). Lipotoxicity in macrophages: Evidence from diseases associated with the metabolic syndrome. Biochim. Biophys. Acta.

[43] Dasu M.R., Jialal I. (2011). Free fatty acids in the presence of high glucose amplify monocyte inflammation via Toll-like receptors. Am. J. Physiol. Endocrinol. Metab..

[44] Shi H., Kokoeva M.V., Inouye K., Tzameli I., Yin H., Flier J.S. (2006). TLR4 links innate immunity and fatty acid-induced insulin resistance. J. Clin. Invest..

[45] Tripathy D., Mohanty P., Dhindsa S., Syed T., Ghanim H., Aljada A., Dandona P. (2003). Elevation of free fatty acids induces inflammation and impairs vascular reactivity in healthy subjects. Diabetes.

[46] Schilling J.D., Machkovech H.M., He L., Sidhu R., Fujiwara H., Weber K., Ory D.S., Schaffer J.E. (2013). Palmitate and lipopolysaccharide trigger synergistic ceramide production in primary macrophages. J. Biol. Chem..

[47] Sitrin R.G., Sassanella T.M., Petty H.R. (2011). An obligate role for membrane-associated neutral sphingomyelinase activity in orienting chemotactic migration of human neutrophils. Am. J. Respir. Cell Mol. Biol..

[48] Niwa M., Kozawa O., Matsuno H., Kanamori Y., Hara A., Uematsu T. (2000). Tumor necrosis factor-α-mediated signal transduction in human neutrophils: Involvement of sphingomyelin metabolites in the priming effect of TNF-α on the fMLP-stimulated superoxide production. Life Sci..

[49] Hinkovska-Galcheva V., Boxer L., Mansfield P.J., Schreiber A.D., Shayman J.A. (2003). Enhanced phagocytosis through inhibition of de novo ceramide synthesis. J. Biol. Chem..

[50] Seumois G., Fillet M., Gillet L., Faccinetto C., DPSmet C., Francois C., Dewals B., Oury C., Vanderplasschen A., Lekeux P. (2007). De novo C16- and C24-ceramide generation contributes to spontaneous neutrophil apoptosis. J. Leukoc. Biol..

[51] Zhang Y., Li X., Grassme H., Doring G., Gulbins E. (2010). Alterations in ceramide concentration and pH determine the release of reactive oxygen species by Cftr-deficient macrophages on infection. J. Immunol..

[52] Sassi F., Tamone C., D’Amelio P. (2018). Vitamin D: Nutrient, Hormone, and Immunomodulator. Nutrients.

[53] Sly L.M., Lopez M., Nauseef W.M., Reiner N.E. (2001). 1alpha,25-Dihydroxyvitamin D3-induced monocyte antimycobacterial activity is regulated by phosphatidylinositol 3-kinase and mediated by the NADPH-dependent phagocyte oxidase. J. Biol. Chem..

[54] El-Sharkawy A., Malki A. (2020). Vitamin D Signaling in Inflammation and Cancer: Molecular Mechanisms and Therapeutic Implications. Molecules..

[55] Leventis P., Patel S. (2008). Clinical aspects of vitamin D in the management of rheumatoid arthritis. Rheumatology.

[56] Mittal M., Siddiqui M.R., Tran K., Reddy S.P., Malik A.B. (2014). Reactive oxygen species in inflammation and tissue injury. Antioxid. Redox Signal..

[57] Lacy P., Stow J.L. (2011). Cytokine release from innate immune cells: Association with diverse membrane trafficking pathways. Blood.

[58] Subramanian K., Bergman P., Henriques-Normark B. (2017). Vitamin D Promotes Pneumococcal Killing and Modulates Inflammatory Responses in Primary Human Neutrophils. J. Innate Immun..

