# Supplementary material for: Differential Modulation of 25-hydroxycholecalciferol on Innate Immunity of Broiler Breeder Hens
Source: Animals (Basel). 2021 Jun 10;11(6):1742. doi: 10.3390/ani11061742 (PMC8230489; doi:10.3390/ani11061742)
Supplement: Supplementary file 1 [file animals-11-01742-s001.zip › animals-1251186-supplementary.pdf]

Supplementary results

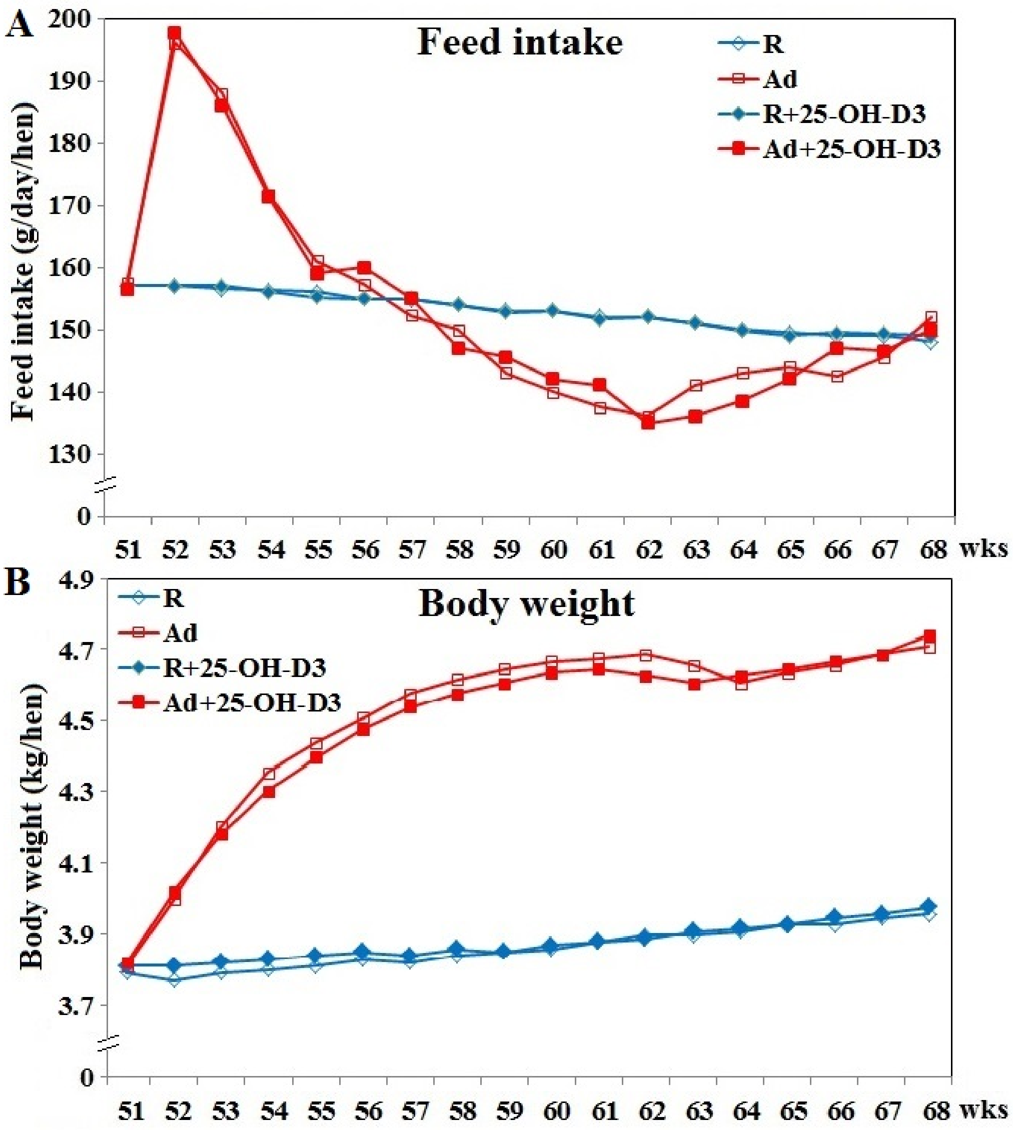

Supplemental Figure S1. Effects of dietary supplementation of 25-OH-D_3 on feed intake and body weight of broiler breeder hens provided with restricted or ad libitum feed intake. At age of 51 weeks, 48 hens of the flock was continued with breeder recommended restricted feeding protocol (Restricted group, R) and another 48 birds were allowed sufficient feed for consumption to appetite (Ad libitum group, Ad). A half of R-hens and Ad-hens were provided with a basal breeder diet with additional supplementation of 25-hydroxycholecalciferol (25-OH-D_3, 69 µg/kg feed).
